# Spatial correlated games

**DOI:** 10.1098/rsos.171361

**Published:** 2017-11-15

**Authors:** Ramón Alonso-Sanz

**Affiliations:** Technical University of Madrid, ETSIAAB, Department of Statistics and GSC, C. Universitaria, Madrid 28040, Spain

**Keywords:** spatial, correlation, games

## Abstract

This article studies correlated two-person games constructed from games with independent players as proposed in Iqbal *et al.* (2016 *R. Soc. open sci.* **3**, 150477. (doi:10.1098/rsos.150477)). The games are played in a collective manner, both in a two-dimensional lattice where the players interact with their neighbours, and with players interacting at random. Four game types are scrutinized in iterated games where the players are allowed to change their strategies, adopting that of their best paid mate neighbour. Particular attention is paid in the study to the effect of a variable degree of correlation on Nash equilibrium strategy pairs.

## 1. Introduction

This paper considers the four two-person (*A* and *B*), 2×2 non-zero-sum game types defined by the pay-off matrices given in table 1. Namely, the Prisoner’s Dilemma (PD), the Hawk–Dove (HD), the Samaritan’s Dilemma (SD) and the Battle of the Sexes (BOS), whose interpretation is described below.

**Table 1.** Four game types. From left to right: Prisoner’s Dilemma (PD), Hawk–Dove (HD), Samaritan’s Dilemma (SD), Battle of the Sexes (BOS).

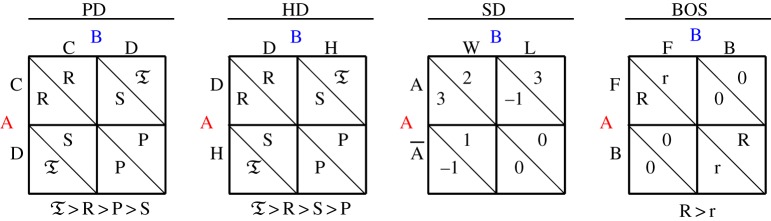

In the PD game, both players may choose either to cooperate (*C*) or to defect (*D*). Mutual cooperators each scoring the *reward* *R*; mutual defectors score the *punishment* *P*; and *D* scores the *temptation* T against *C*, who scores *S* (*sucker*’s pay-off) in such an encounter. In the PD, it is: T>R>P>S. In this study, the PD pay-off values will be T=5, *R*=3, *P*=2 and *S*=1. The PD with these pay-offs will be referred to as PD(5,3,2,1).

In the HD game, the structure of the pay-offs matrices is similar to that in the PD, but in the HD it is *P*<*S* instead of *P*>*S* as in the PD. In this study, the HD pay-off values will be T=3, *R*=2, *P*=−1 and *S*=0. The HD with these pay-offs will be referred to as HD(3,2,0,−1).

In the SD game, the charity player A may choose Aid/No Aid, whereas the beneficiary player B may choose Work/Loaf. The Samaritan’s dilemma arises in the act of charity. The charity player wants to help (Aid) people in need. However, the beneficiary may simply rely on the handout (Loaf) rather than try to improve their situation (Work). This is not anticipated by the charity player. Many people may have experienced this dilemma when confronted with people in need. Although there is a desire to help them, there is the recognition that a handout may be harmful to the long-run interests of the recipient [1–5]. Following the references [6–8], we adopt here the pay-off matrices given in its corresponding panel in table 1, and the SD with these pay-offs will be referred to as SD(3,2,1,−1).

In the so-called BOS game, the rewards *R*>*r*>0 quantify the preferences in a conventional couple fitting the traditional stereotypes: The male player A prefers to attend a *F*ootball match, whereas the female player B prefers to attend a *B*allet performance. Both players hope to *coordinate* their choices, but the *conflict* is also present because their preferred activities differ [9,10]. In this study, the BOS pay-off values will be *R*=3, *r*=1. The BOS with these pay-offs will be referred to as BOS(5,1).

The PD and HD games are symmetric, i.e. the pay-off matrices of both players coincide after transposition, whereas the SD and the BOS games are not symmetric. In symmetric games, the role of both players are somehow interchangeable, whereas in asymmetric games every player has to be studied separately. This issue is to be taken into account all across this study, but particularly in §5.

This paper studies the game-types under scrutiny interacting in a collective manner; either with players connected in a spatially structured manner (§3) or with players randomly connected (§4). Collective games on networks have long been studied previously [11,12]. The novelty of this study lies in the consideration of the mechanism for correlating independent strategies given in [13], and contextualized here in §2.

## 2. Independent players and correlated games

In the somehow canonical approach to game theory, both players choose their strategies independently of each other. In an alternative approach, an external (probabilistic) mechanism sends a signal to each player, so that, in principle, the players do not have any active role. Both approaches, as well as a mechanism for combining them, are featured in this section.

### 2.1 Games with independent players

In conventional games, both players decide independently their probabilistic strategies **x**=(*x*,1−*x*)′ and **y**=(*y*,1−*y*)′, which give rise to the joint probability distribution ***Π***=**xy**′. As a result, in a game with **P**_*A*_ and **P**_*B*_ pay-off matrices, the expected pay-offs (*p*) of both players are (⊙ indicates element-by-element matrix multiplication, 1′=(1,1)):

2.1 $$p_{A}(x,y)=1^{\prime }{\text{P}}_{A}⊙\Pi 1={\text{x}}^{\prime }{\text{P}}_{A}\text{y},\;p_{B}(x,y)=1^{\prime }{\text{P}}_{B}⊙\Pi 1={\text{x}}^{\prime }{\text{P}}_{B}\text{y}.$$

The strategy pair (**x**, **y**), referred to here as (*x*, *y*), is in Nash equilibrium (NE), if *x* is the best response to *y* and *y* is the best response to *x*. In the PD game, mutual defection, i.e. x*=y*=0, is the only pair of strategies in NE. The HD game has three strategy pairs in NE, two of them are given by the pure strategies (x*=1,y*=0≡(*D*,*H*)) and (x*=0,y*=1≡(*H*,*D*)), whereas the third NE in achieved with mixed strategies, which in the particular case of the HD(3,2,0,−1) considered here becomes $x^{*}=y^{*}=\frac{1}{2}$, leading to *p*_*A*,*B*_=1. Note that (x*=y*=0≡(*H*,*H*)) is not in NE in the HD game. The SD game has only one NE, which in the particular case of the SD(3,2,1,−1) considered here becomes: $\left(x^{*}=\frac{1}{2},y^{*}=\frac{1}{5}\right)$, leading to (*p*_A_=−0.2, *p*_B_=1.5). The BOS game has three strategy pairs in NE, two of them are given by the pure strategies (x*=y*=1≡(*F*,*F*)) and (x*=y*=0≡(*B*,*B*)), whereas the third NE in achieved with the mixed strategies (x*=*R*/(*R*+*r*),y*=*r*/(*R*+*r*)), leading to *p*_*A*,*B*_=*Rr*/(*R*+*r*)<*r*.

Social welfare (SW) functions may be envisaged as summarizing some particular conception of the *common good* [14]. In its simplest form, SW solutions maximize the sum of the pay-offs of both players. In the games studied here, only (1,1) is the SW solution in the HD(3,2,0,−1) and the SD(3,2,1,−1); in the PD(5,3,2,1), (1,1), (1,0), (0,1) are SW solutions, although only (1,1) is pay-offs balanced; in the BOS(5,1), both (1,1) and (0,0) are SW solutions.

### 2.2 Correlated games

In a different game scenario, that of correlated games, an *external* probability distribution $\Pi =\left(\begin{array}{cc} \pi_{11} & \pi_{12}\\ \pi_{21} & \pi_{22} \end{array}\right)$ assigns probability to every combination of player choices [10], giving rise to the expected pay-offs *p*_*A*_(***Π***)=1′**P**_*A*_⊙***Π***1, *p*_*B*_(***Π***)=1′**P**_*B*_⊙***Π***1.

Non-factorizable ***Π*** may be generated from independent strategies (*x*,*y*) as with the ad hoc method based on an external parameter *k*∈[0,1] given in [13], and shown as follows:

2.2 $$\begin{array}{rl} \pi_{11} & \;={\left(2k-1\right)}^{2}xy\;\pi_{12}=(1-k)x(1-y)+k(1-x)y\\ \text{and}\;\;\pi_{21} & \;=(1-k)(1-x)y+kx(1-y)\;\pi_{22}=(1-x)(1-y)+4k(1-k)xy \end{array}\}$$

Equations (2.3) give the values of the elements of ***Π*** from equations (2.2) for three relevant values of *k*. Note that *k*=1 interchanges the *k*=0 values of *π*_12_ and *π*_21_, whereas those of *π*_11_ and *π*_22_ remain unaltered. Also relevant is that if $x=y=\frac{1}{2}$, ***Π*** is uniform (all its elements equal to $\frac{1}{4}$) for *k*=0 and *k*=1, but for $k=\frac{1}{2},$ it is $\pi_{11}=0,\pi_{12}=\pi_{21}=\frac{1}{4},\pi_{22}=\frac{2}{4}$. As a result, in a balanced $x=y=k=\frac{1}{2}$ scenario, the player B is privileged in the KBOS game. Thus, following with the male–female stereotypes, a male modeller would describe the BOS game assigning player B to the female, whereas the female modeller would reverse such role assignments.

2.3a $$\begin{array}{rl} k & \;=0:\;\pi_{11}=xy,\;\pi_{12}=x(1-y),\;\pi_{21}=(1-x)y,\;\pi_{22}=(1-x)(1-y), \end{array}$$

2.3b $$\begin{array}{rl} k & \;=\frac{1}{2}:\;\pi_{11}=0,\;\pi_{12}=\pi_{21}=\frac{\left(x+y-2xy\right)}{2},\;\pi_{22}=(1-x)(1-y)+xy \end{array}$$

2.3c $$\begin{array}{rl} \text{and}\;k & \;=1:\;\pi_{11}=xy,\;\pi_{12}=(1-x)y,\;\pi_{21}=x(1-y),\;\pi_{22}=(1-x)(1-y) \end{array}$$

the following equations give the elements of *Π* from equations (2.2) for relevant values of *x* and *y*.

2.4a $$\begin{array}{rl} \pi_{11}^{\left(1.0,1.0\right)} & \;={\left(2k-1\right)}^{2},\;\pi_{12}^{\left(1.0,1.0\right)}=\pi_{21}^{\left(1.0,1.0\right)}=0,\;\pi_{22}^{\left(1.0,1.0\right)}=4k(1-k), \end{array}$$

2.4b $$\begin{array}{rl} \pi_{11}^{\left(1.0,0.0\right)} & \;=0,\;\pi_{12}^{\left(1.0,0.0\right)}=1-k,\;\pi_{21}^{\left(1.0,0.0\right)}=k,\;\pi_{22}^{\left(1.0,0.0\right)}=0, \end{array}$$

2.4c $$\begin{array}{rl} \pi_{11}^{\left(0.0,1.0\right)} & \;=0,\;\pi_{12}^{\left(0.0,1.0\right)}=k,\;\pi_{21}^{\left(0.0,1.0\right)}=1-k,\;\pi_{22}^{\left(0.0,1.0\right)}=0, \end{array}$$

2.4d $$\begin{array}{rl} \pi_{11}^{\left(0.0,0.0\right)} & \;=\pi_{12}^{\left(0.0,0.0\right)}=\pi_{21}^{\left(0.0,0.0\right)}=0,\;\pi_{22}^{\left(0.0,0.0\right)}=1 \end{array}$$

2.4e $$\begin{array}{rl} \text{and}\;\pi_{11}^{\left(0.5,0.5\right)} & \;=\frac{1}{4}-k(1-k),\;\pi_{12}^{\left(0.5,0.5\right)}=\pi_{21}^{\left(0.5,0.5\right)}=\frac{1}{4},\;\pi_{22}^{\left(0.5,0.5\right)}=\frac{1}{4}+k(1-k). \end{array}$$

From the joint probabilities given in equations (2.4), the pay-offs in a KPD(5,3,2,1) with pure strategies and *x*=*y*=0.5 are given in the following equations, and plotted in figure 3*a*:

2.5a $$\begin{array}{rl} p_{A}^{\left(1.0,1.0\right)} & \;=p_{B}^{\left(1.0,1.0\right)}=4k^{2}-4k+3, \end{array}$$

2.5b $$\begin{array}{rl} p_{A}^{\left(1.0,0.0\right)} & \;=1+4k,\;p_{B}^{\left(1.0,0.0\right)}=5-4k, \end{array}$$

2.5c $$\begin{array}{rl} p_{A}^{\left(0.0,1.0\right)} & \;=5-4k,\;p_{B}^{\left(0.0,1.0\right)}=1+4k, \end{array}$$

2.5d $$\begin{array}{rl} p_{A}^{\left(0.0,0.0\right)} & \;=p_{B}^{\left(0.0,0.0\right)}=2 \end{array}$$

2.5e $$\begin{array}{rl} \text{and}\;p_{A}^{\left(0.5,0.5\right)} & \;=p_{B}^{\left(0.5,0.5\right)}=\frac{1}{4}(11-k(1-k)). \end{array}$$

**Figure 1.**
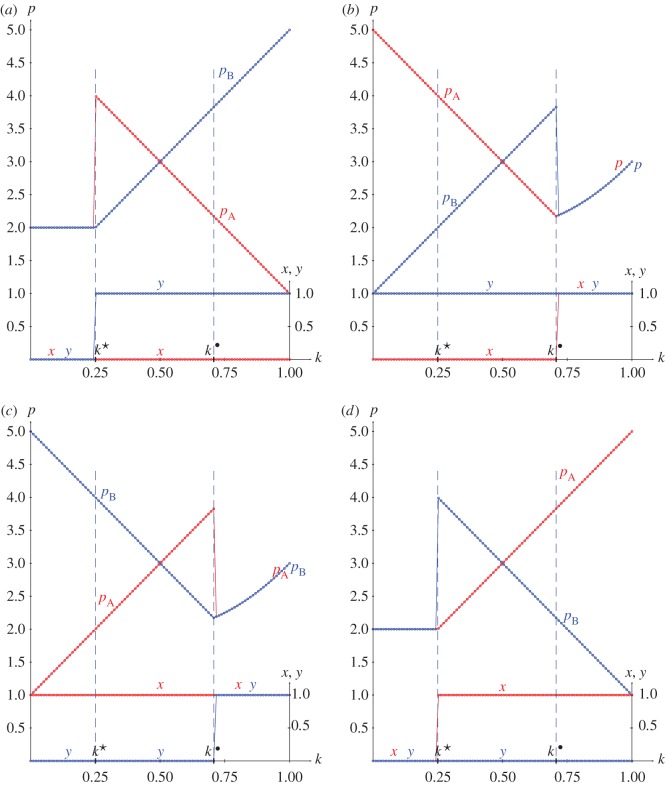
Best responses to pure strategies in the KPD(5,3,2,1). (*a*) *x*=0, (*b*) *y*=1, (*c*) *x*=1, (*d*) *y*=0.

Figure 1 shows the best responses to pure strategies in the KPD(5,3,2,1). Figure 1*a*,*b* proves, respectively, that the strategy pairs (0,1) and (1,0) are in NE in the (*k*^★^,*k*^•^) interval. The *k*^★^-threshold is achieved in the intersection of $p_{A,B}^{0,0}=2$ and $p_{A}^{1,0}=p_{B}^{0,1}=1+4k$, thus $k^{⋆}=\frac{1}{4}$, whereas the *k*^•^-threshold is achieved in the intersection of $p_{A}^{0,1}=p_{B}^{1,0}=5-4k$ and $p_{A,B}^{1,1}=4k^{2}-4k+3$, thus $k^{∙}=1/\sqrt{2}=0.707$.

From the joint probabilities given in equations (2.4), the pay-offs in a KHD(3,2,−1,0) with pure strategies and *x*=*y*=0.5 are given in the following equations, and plotted in figure 4*a*.

2.6a $$\begin{array}{rl} p_{A}^{\left(1.0,1.0\right)} & \;=p_{B}^{\left(1.0,1.0\right)}=12k^{2}-12k+2, \end{array}$$

2.6b $$\begin{array}{rl} p_{A}^{\left(1.0,0.0\right)} & \;=3k,\;p_{B}^{\left(1.0,0.0\right)}=3(1-k), \end{array}$$

2.6c $$\begin{array}{rl} p_{A}^{\left(0.0,1.0\right)} & \;=3(1-k),\;p_{B}^{\left(0.0,1.0\right)}=3k, \end{array}$$

2.6d $$\begin{array}{rl} p_{A}^{\left(0.0,0.0\right)} & \;=p_{B}^{\left(0.0,0.0\right)}=-1 \end{array}$$

2.6e $$\begin{array}{rl} \text{and}\;p_{A}^{\left(0.5,0.5\right)} & \;=p_{B}^{\left(0.5,0.5\right)}=1-3k(1-k). \end{array}$$

From the joint probabilities given in equations (2.4), the pay-offs in a KSD(3,2,−1,0) with pure strategies and *x*=*y*=0.5 are given in the following equations, and plotted in figure 6*a*.

2.7a $$\begin{array}{rl} p_{A}^{\left(1.0,1.0\right)} & \;=12k^{2}-12k+3,\;p_{B}^{\left(1.0,1.0\right)}=8k^{2}-8k+2, \end{array}$$

2.7b $$\begin{array}{rl} p_{A}^{\left(1.0,0.0\right)} & \;=-1,\;p_{B}^{\left(1.0,0.0\right)}=3-2k, \end{array}$$

2.7c $$\begin{array}{rl} p_{A}^{\left(0.0,1.0\right)} & \;=-1,\;p_{B}^{\left(0.0,1.0\right)}=1+2k, \end{array}$$

2.7d $$\begin{array}{rl} p_{A}^{\left(0.0,0.0\right)} & \;=p_{B}^{\left(0.0,0.0\right)}=0 \end{array}$$

2.7e $$\begin{array}{rl} \text{and}\;p_{A}^{\left(0.5,0.5\right)} & \;=\frac{1}{4}-3k(1-k),\;p_{B}^{\left(0.5,0.5\right)}=\frac{3}{2}-2k(1-k). \end{array}$$

In the KSD(3,2,−1,0) it is, *p*_A_=((3(2*k*−1)^2^+2)*y*−1)*x*−*y*, *p*_B_=((2(2*k*−1)^2^−4)*x*+1+2*k*)*y*+(3−2*k*)*x*. Consequently, the strategy pairs in NE in the KSD(3,2,−1,0) are given in (2.8), where the threshold k*=0.89 emerges from the *x*≤1 restraint applied to *x*. Before k*, it is

2.8 $$\begin{array}{rl} & p_{A}=-\frac{1}{2+3{\left(2k-1\right)}^{2}},\;p_{B}=\frac{\left(3-2k)(1+2k\right)}{4-2{\left(2k-1\right)}^{2}}.\\ & \text{NE}(k):\begin{array}{ll} \left(x=\frac{1+2k}{4-2{\left(2k-1\right)}^{2}},y=\frac{1}{2+3{\left(2k-1\right)}^{2}}\right) & k\leq k^{*}=0.89\\ \left(x=1,y=1\right) & k\geq k^{*}=0.89 \end{array}\}. \end{array}$$

**Figure 2.**
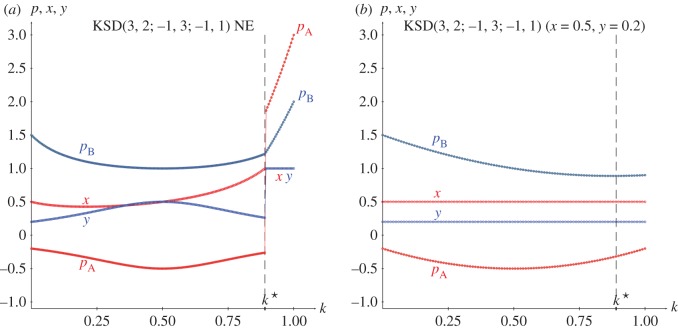
The KSD(3,2,−1,0). (*a*) Strategies and pay-offs in NE. (*b*) Pay-offs with (*x*=0.5,*y*=0.2).

Figure 2*a* shows the strategies and pay-offs in NE in a KSD(3,2,−1,0) for variable *k*. Figure 2*b* shows pay-offs in a KSD(3,2,−1,0) with (*x*=0.5,*y*=0.2). It is remarkable that the pay-offs in the latter scenario do not differ very much from that in NE, particularly in the case of *p*_A_.

From the joint probabilities given in equations (2.4), the pay-offs in a KBOS(5,1) with pure strategies and *x*=*y*=0.5 are given in the following equations, and plotted in figure 7*a*.

2.9a $$\begin{array}{rl} p_{A}^{\left(1.0,1.0\right)} & \;=16k^{2}-16k+5,\;p_{B}^{\left(1.0,1.0\right)}=-16k^{2}+16k+1, \end{array}$$

2.9b $$\begin{array}{rl} p_{A}^{\left(1.0,0.0\right)} & \;=p_{B}^{\left(1.0,0.0\right)}=p_{A}^{\left(0.0,1.0\right)}=p_{B}^{\left(0.0,1.0\right)}=0, \end{array}$$

2.9c $$\begin{array}{rl} p_{A}^{\left(0.0,0.0\right)} & \;=1,\;p_{B}^{\left(0.0,0.0\right)}=5 \end{array}$$

2.9d $$\begin{array}{rl} \text{and}\;p_{A}^{\left(0.5,0.5\right)} & \;=\frac{3}{2}-4k(1-k),\;p_{B}^{\left(0.5,0.5\right)}=\frac{3}{2}+4k(1-k). \end{array}$$

Iqbal *et al.* [13] give a second method of constructing non-factorizable ***Π*** from independent strategies (*x*,*y*). It departs from the fact that in factorizable ***Π*** it is *π*_11_=*xy*, *π*_12_=*x*−*π*_11_, *π*_21_=*y*−*π*_11_, *π*_22_=1+*π*_11_−(*x*+*y*). Then, it is proposed just to alter the form of *π*_11_=*xy*, maintaining those of the other three elements of ***Π*** as functions of *π*_11_. It is concluded in [13] that *π*_11_(*x*,*y*)<*xy* is the only restriction to be imposed on *π*_11_(*x*,*y*) in order to make sure that all the elements of ***Π*** are in the [0,1] interval and sum to 1.0. The authors propose *π*_11_=(*xy*)^2^ and *π*_11_=*x*^2^*y*^3^ as examples. But *π*_11_(*x*,*y*)<*xy* does not suffice to make sure that *π*_22_=1+*π*_11_−(*x*+*y*) is non-negative. To prove this, let us consider the particular case of *x*=*y*, i.e. *π*_22_=1+*π*_11_−2*x*: With *π*_11_=*x*^4^, *π*_22_ is negative if 0.554<*x*<1.0, and with *π*_11_=*x*^5^, *π*_22_ is negative if 0.519<*x*<1.0.

## 3. Spatial games

In the spatial version of the two-person games we deal with, each player occupies a site (*i*,*j*) in a two-dimensional *N*×*N* lattice. The *A* and *B* players alternate in the site occupation in a chessboard form, so that every player is surrounded by four partners (*A*-*B*, *B*-*A*), and four mates (*A*-*A*, *B*-*B*). The game is played in the cellular automata (CA) manner, i.e. with uniform, local and synchronous interactions [15]. In this way, every player (*i*,*j*) plays with his four adjacent partners, so that his pay-off at time step *T*, namely $p_{i,j}^{\left(T\right)}$, is the sum over these four interactions. The evolution is ruled by the (deterministic) imitation of the best paid neighbour, so that in the next generation, every generic player (*i*,*j*) will adopt the probabilities of his mate player (*k*,*l*) with the highest pay-off among their mate neighbours. Table 2 shows a simple example with the PD(5,3,2,1) game where initially every player cooperates (*x*=*y*=1), except the defector (*x*=0) player A located in the (3,4) cell. Thus at *T*=1, the defector player A gets the *p*=20 pay-off instead of the common *p*=12 pay-off. The imitation mechanism spreads the *x*_*A*_=1 defection across the player A cells, whereas player B cooperation remains unaltered as no player B defects.

**Table 2.** A simple example in the spatial PD(5,3,2,1) scenario. Far left: The (A,B) chessboard. Centre: Initially every player cooperates, except the defector player A located in the (3,4) cell. Far right: Probabilities and pay-offs at *T*=2.

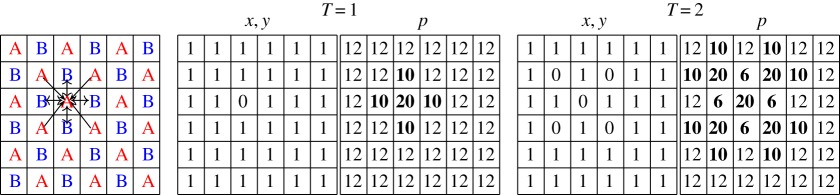

All the simulations in this section are run in an *N*=200 lattice with periodic boundary conditions and initial random assignment of the probability values sampled from a uniform distribution in the [0,1] interval. Thus, initially: $\bar{x}≃0.5$ and $\bar{y}≃0.5$. As a rule, the results regarding player A are shown in red, and those regarding player B are shown in blue. The computations have been performed by a double precision Fortran code run on a mainframe.

**Figure 3.**
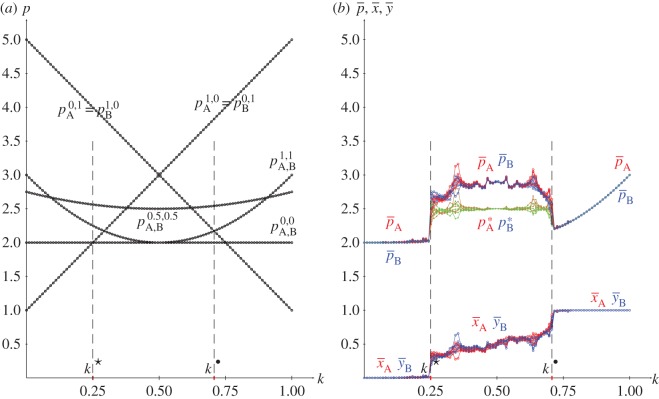
The KPD(5,3,2,1) with variable *k*. (*a*) Pay-offs in two-person games. (*b*) Mean pay-offs and mean values of *x* and *y* in five spatial simulations at *T*=200.

Figure 3 deals with spatial simulations of the PD(5,3,2,1) with joint probabilities generated according to (2.2). Figure 3*b* shows the mean pay-offs ($\bar{p}$) and mean values of *x* and *y* at *T*=200 starting from five different random assignments of *x* and *y*. Mutual defection (*x*=*y*=0) arises below the lower *k*^★^=0.25 threshold and mutual cooperation (*x*=*y*=1) beyond the higher *k*^•^=0.707 threshold. In the (*k*^★^,*k*^•^) transition interval, where both (1,0) and (0,1) are in NE, $\bar{x}$ and $\bar{y}$ are fairly similar, increasing their values from 0.0 to 1.0 as *k* increases from *k*^★^ up to *k*^•^; the mean pay-offs of both players in turn are fairly similar, reaching values not far from *R*=3. With the more sophisticated method of correlating independent probability distributions presented in [16], referred to here as EWL, the transition interval from mutual defection up to mutual cooperation in the PD is shorter and a strategy pair in NE providing the pay-off of mutual cooperation appears with lower degree of correlation (entanglement in the quantum approach implemented by the EWL method). In the PD(5,3,2,1) studied here, the thresholds of the correlation parameter applying the EWL method (referred to here as *k*_*q*_) in a 0.0 up to 1.0 normalized scale are $k_{q}^{⋆}=0.333$ and $k_{q}^{∙}=0.500$ [17].

Figure 3*b* shows also the mean-field pay-offs (p*) achieved in a single hypothetical two-person game with players adopting the mean probabilities appearing in the spatial dynamic simulation, namely with joint probability matrix

3.1 $$\Pi^{⋆}=\left(\begin{array}{cc} {\left(2k-1\right)}^{2}\bar{x}\;\bar{y} & (1-k)\bar{x}(1-\bar{y})+k(1-\bar{x})\bar{y}\\ \left(1-k)(1-\bar{x})\bar{y}+k\bar{x}(1-\bar{y}\right) & (1-\bar{x})(1-\bar{y})+4k(1-k)\bar{x}\;\bar{y} \end{array}\right).$$

The mean-field pay-offs (coloured brown for player A, green for player B) fully coincide with the actual mean pay-offs out of the transition interval, but underestimate them in the transition interval. The lack of coincidence of both mean-field and actual mean pay-offs is due to spatial effects that will be illustrated here when addressing the BOS game (figures 9 and 8).

**Figure 4.**
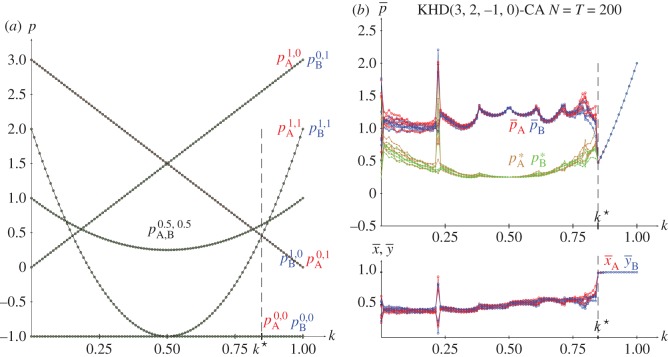
The KHD(3,2,0,−1) with variable *k*. (*a*) Pay-offs in two-person games. (*b*) Mean pay-offs and mean values of *x* and *y* in five spatial simulations at *T*=200.

Figure 4*b* shows the results in five spatial simulations of the HD(3,2,0,−1) at *T*=200. Spatial effects arise before *k*^★^ so that the mean-field approaches underestimate the actual mean pay-offs as in the spatial simulations of the PD. After *k*^★^, the spatial simulations detect (1,1) as the unique NE, so that both pay-offs increase their values according to *p*=12*k*^2^−12*k*+2 up to *p*=2.0 at *k*=1.0. The *k*^★^ threshold appears from the intersection of *p*^(1.0,1.0)^ and *p*^(1.0,0.0)^_*B*_, given in equations (2.6a) and (2.6b), thus *k*^★^=0.848.

**Figure 5.**
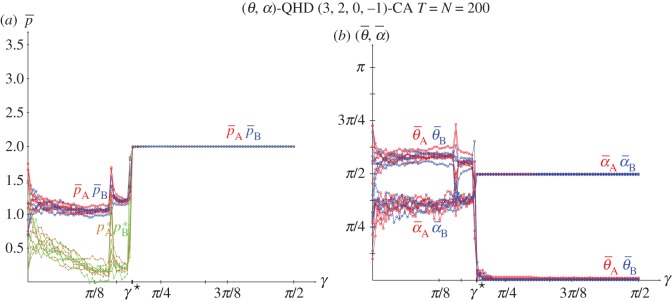
The spatial Quantum HD(3,2,0,−1) with variable entanglement factor *γ*. (*a*) Mean pay-offs. (*b*) Mean quantum parameter values. Five simulations at *T*=200.

No results on the spatial simulations of the HD using the EWL correlation method have been reported elsewhere, so figure 5 is included in this article. Again, as stressed above regarding the PD, the outcome of mutual cooperation (Dove in the HD) emerges before with the EWL method: $k_{q}^{⋆}=0.392<k_{c}^{⋆}=0.848$. Note in figure 5*a* that spatial effects also arise in spatial simulations using the EWL method before $k_{q}^{⋆}$, so that the mean-field estimates (*p*^★^) also underestimate the actual mean pay-offs ($\bar{p}$) in the QHD before the $k_{q}^{⋆}$ threshold.

**Figure 6.**
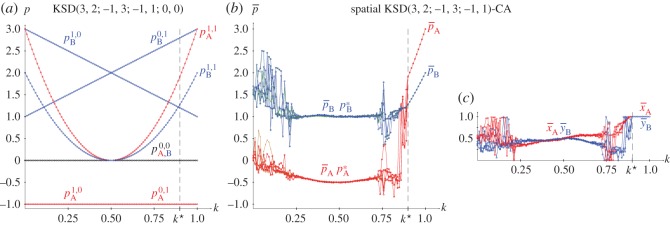
The KSD(3,2,1,−1) with variable *k*. (*a*) Pay-offs in two-person games. (*b*,*c*) Mean pay-offs and mean values of *x* and *y* in five spatial simulations at *T*=200.

Figure 6*b*,*c* show the results in five spatial simulations of a KSD(3,2,−1,0) at *T*=200. As the SD has only one NE regardless of *k*, (i) the results shown in the spatial simulation mimic those corresponding to NE in two-person games shown in figure 2*a*, and (ii) no spatial effects arise so that both mean-field and actual pay-offs coincide for every *k*. In spatial simulations of the SD using the EWL correlation method [6] it is $k_{q}^{•}=0.500>k_{c}^{•}=0.890$.

**Figure 7.**
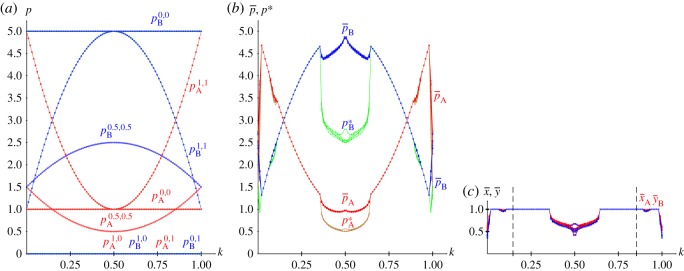
The KBOS(5,1) with variable *k*. (*a*) Pay-offs in two-person games. (*b*,*c*) Five spatial simulations at *T*=200. (*b*) Mean pay-offs and mean-field approaches, (*c*) mean values of *x* and *y*.

Figure 7*b*,*c* show the results in five spatial simulations of the KBOS(5,1) at *T*=200. Owing to the particular structure of the BOS game, where both *π*_12_ and *π*_21_ are irrelevant, the graphs in these panels are symmetric around *k*=0.5. The general form of the pay-offs (figure 7*b*) correspond to that of $\bar{x}=\bar{y}=1$, diminishing close to *k*=0.5 (figure 7*c*) where notable spatial effects arise, and particularly close to the extreme values of *k*, both 0.0 and 1.0. The output of the spatial simulations of the BOS using the EWL correlation method notably differ from that shown in figure 7 [18]. Let us say that the BOS game proves to be a highly challenging game.

**Figure 8.**
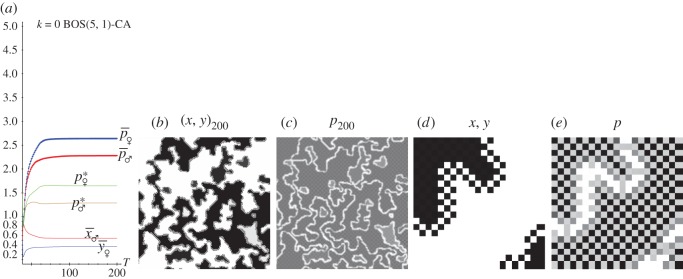
The spatial KBOS(5,1) with *k*=0.0. (*a*) Dynamics up to *T*=200. (*b*,*c*) Patterns of the full 200×200 lattice at *T*=200. (*d*,*e*) Zooms of the 20×20 central area.

**Figure 9.**
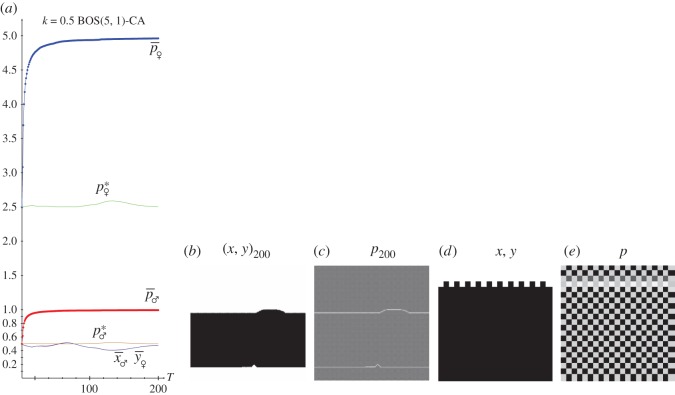
The spatial KBOS(5,1) with *k*=0.5. (*a*) Dynamics up to *T*=200. (*b,c*) Patterns of the full 200×200 lattice at *T*=200. (*d,e*) Zooms of the 20×20 central area.

Figures 8 and 9 deal with simulations of the KBOS(5,1). The former with *k*=0.0, the latter with *k*=0.5. In panel *a* of both figures, the dynamics up to *T*=200; in panels *b* and *c*, the patterns of pay-offs and probabilities at *T*=200 and in panels *d* and *e*, zooms of the 20×20 central region of the full patterns. In both scenarios, the dynamics induced by the imitation of the best paid neighbour implemented here actuates in a straightforward manner, so that the permanent regime is achieved very soon. This applies not only to the BOS game but in a general manner, regardless of the game under scrutiny.

The patterns of the pay-offs and probabilities shown in figure 8*b,c* are enhanced by the zooms of a small central region in figure 8*d,e*. The general patterns are featured by regions of black-marked clusters where *x*=*y*=1.0 and white-marked clusters where *x*=*y*=0.0. The emergence of these well-defined spatial structures explain why the mean-field pay-off fails to estimate the actual mean pay-off, as shown in figure 8*a*. The pattern of probabilities at *T*=200 shown in figure 9 for *k*=0.5 turns out particularly surprising as two horizontal compact bands with (*x*=*y*=0.0) (figure 9*a*, upper and lower panels) and one with (*x*=*y*=1.0) emerge. This dramatic spatial structure lies in the origin of the discrepancy between the mean-field and the actual mean pay-offs shown in figure 9*a*. In figures 8 and 9, the (*x*=*y*=0.0) and (*x*=*y*=1.0) clusters are separated by borders where either (*x*=0.0,*y*=1.0) or (*x*=1.0,*y*=0.0) and consequently the pay-offs of both players are zero, which causes white cell lines in the pay-offs patterns. These clear (almost white) border lines are clearly noticeable in figure 8*b–e*, whereas in figure 9 they are only two not-so-apparently clear horizontal lines, one of them enhanced in the zoom which has been located in the upper transition from *x*=*y*=0.0 to *x*=*y*=1.0.

## 4. Games on random networks

In the simulations of this section, every player is connected at random with four partners and four mates, so that any spatial structure is absent in such random networks. To compare the simulations presented in this section to those based in spatially structured lattices in §3, also 200×200 players interact in the games on networks studied in this section, half of them of type A, the other half of type B.

**Figure 10.**
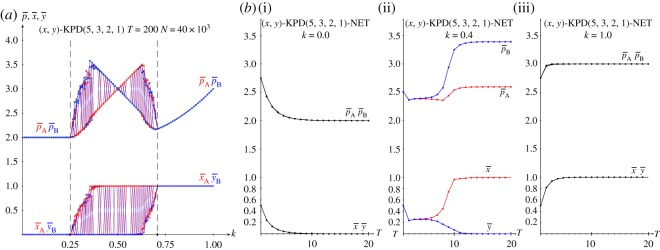
The KPD(5,3,2,1) with variable *k* in network simulations. (*a*) Mean pay-offs and mean values of *x* and *y* at *T*=200 in five simulations. (*b*) Dynamics up to *T*=20 of one of such simulations with *k*=0 (i), *k*=0.4 (ii) and *k*=1.0 (iii).

Figure 10 deals with the KPD(5,3,2,1) game with variable *k* in network simulations. Figure 10*a* shows the mean pay-offs of both players and their mean values of *x* and *y* at *T*=200 in five simulations. Figure 10*b* shows the dynamics in one of such simulations up to *T*=20 for *k*=0.0 (i), *k*=0.4 (ii) and *k*=1.0 (iii).

The overall structure of the graphs in figure 10*a* coincides with that in figure 3*b*. The *k*^★^ and *k*^•^ remain unaltered, with *x*=*y*=0 before *k*^★^ and *x*=*y*=1 after *k*^★^ in both scenarios. At variance with this, the behaviour of the system in the (*k*^★^,*k*^•^) interval varies significantly in figure 10 compared to that in figure 3, as in the network simulation the (1,0) and (0,1) NE emerge with no spatial effects masking them. Panel *b* shows that also in network simulations the dynamics induced by the imitation of the best paid neighbour implemented here also actuates in a straightforward manner, so that the permanent regime is achieved almost immediately for *k*=0.0 and *k*=1.0, and as soon as just passed *T*=10 for *k*=0.4.

**Figure 11.**
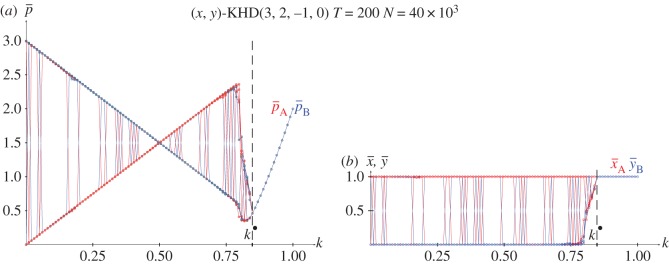
The KHD(3,2,0−1) with variable *k* in five network simulations at *T*=200. (*a*) Mean pay-offs and (*b*) mean values of *x* and *y*.

**Figure 12.**
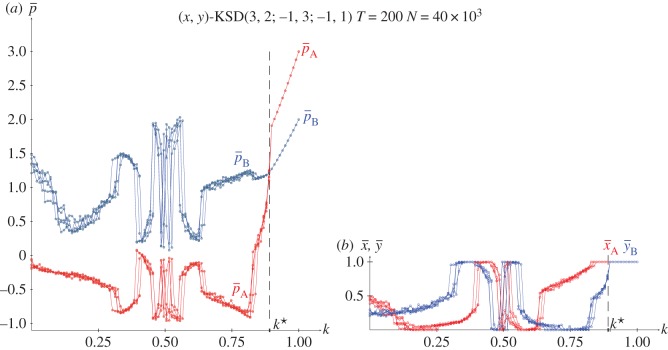
The KSD(3,2,1,−1) with variable *k* in five network simulations at *T*=200. (*a*) Mean pay-offs and (*b*) mean values of *x* and *y*.

**Figure 13.**
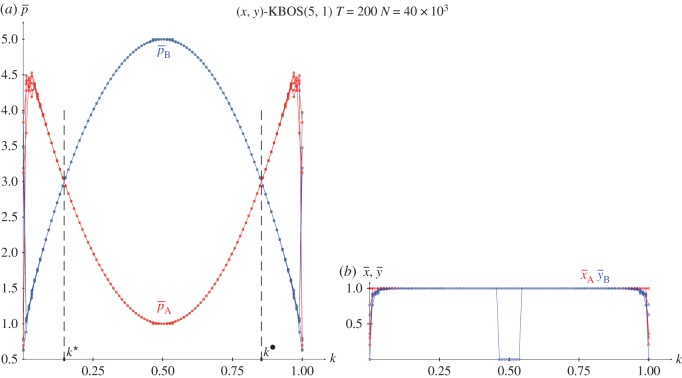
The BOS(5,1) with variable *k* in five network simulations at *T*=200. (*a*) Mean pay-offs and (*b*) mean values of *x* and *y*.

Figures 11–13 show the results with the KHD(3,2,0,−1), KSD(3,2,1,−1) and KBOS(5,1) games with variable *k* in five network simulations at *T*=200. Panel *a* of these figures shows the mean pay-offs of both players, and panel *b*, the mean values of *x* and *y*.

In figure 11, the *k*^•^ threshold and the permanent *x*=*y*=1 regime after *k*^•^ remain unaltered compared to those in figure 4. But before *k*^•^, the KHD system behaves much as the KPD in its transition interval in network simulations: the (1,0) and (0,1) NE emerge with no spatial effects masking them.

In figure 12, the *k*^•^ threshold and the permanent *x*=*y*=1 regime after *k*^•^ remain unaltered compared to those in figure 6. But before *k*^•^, the KSD system shows a kind of helter-skelter oscillations particularly pronounced around *k*=0.5.

The overall structure of the graphs in figure 13 coincides with that in figure 7, so that $\bar{x}=\bar{y}=1$ prevail, except close to the extreme values of *k*, both 0.0 and 1.0. The absence of spatial structure in the network simulations of figure 13 produces crisp pay-offs (and probability) graphs, with no relevant alterations around *k*=0.5 , although in one of the simulations it is $\bar{x}=\bar{y}=0$ rendering $p_{A}^{\left(0.0,0.0\right)}=1,\;p_{B}^{\left(0.0,0.0\right)}=5$ close to *k*=0.5, coincident with $p_{A}^{\left(1.0,1.0\right)}(k=0.5)=1,\;p_{B}^{\left(1.0,1.0\right)}(k=0.5)=5$. In the graphs of pay-offs in figure 13*a* player B overrates player A in the wide interval $\left(k^{⋆}=0.5-\sqrt{2}/4,k^{⋆}=0.5+\sqrt{2}/4\right)$ (with *k*^★^ and *k*^•^ defined at the intersection of the pay-offs given in equations (2.9a)). This indicates a kind of bias of the proposed correlation mechanism that favours player B (already pointed out when commenting on equations (2.3) in §2.2), a characteristic that is also found in the EWL model regarding the BOS game [18]. It is relevant to point out that $\pi_{11}^{\left(1.0,1.0\right)}(k^{⋆})=\pi_{11}^{\left(1.0,1.0\right)}(k^{•})=\pi_{22}^{\left(1.0,1.0\right)}(k^{⋆})=\pi_{22}^{\left(1.0,1.0\right)}(k^{•})=\frac{1}{2}$, leading to the maximum attainable equalitarian pay-off in the BOS: $p_{A}^{\left(1.0,1.0\right)}(k^{⋆})=p_{B}^{\left(1.0,1.0\right)}(k^{⋆})=p_{A}^{\left(1.0,1.0\right)}(k^{•})=p_{B}^{\left(1.0,1.0\right)}(k^{•})=3=\left(R+r\right)/2.$ Note that the maximum attainable equalitarian pay-off in the BOS with independent players is half the previous one, i.e. $p_{A}=p_{B}=\frac{3}{2}=\left(R+r\right)/4$, achieved with $x=y=\frac{1}{2}$, which leads to $\pi_{11}=\pi_{12}=\pi_{21}=\pi_{22}=\frac{1}{4}$.

## 5. Partial strategy updating

**Figure 14.**
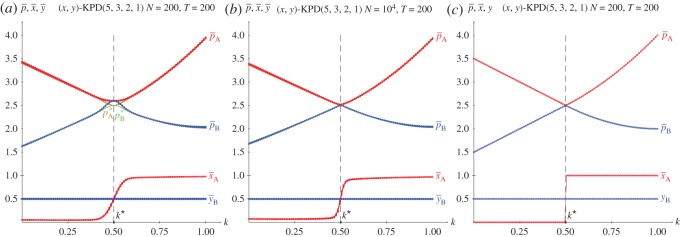
The KPD(5,3,2,1) with variable *k* when only player A updates strategies. Five simulations at *T*=200. (*a*) Spatial simulations, (*b*) games on networks and (*c*) *y*=0.5.

**Figure 15.**
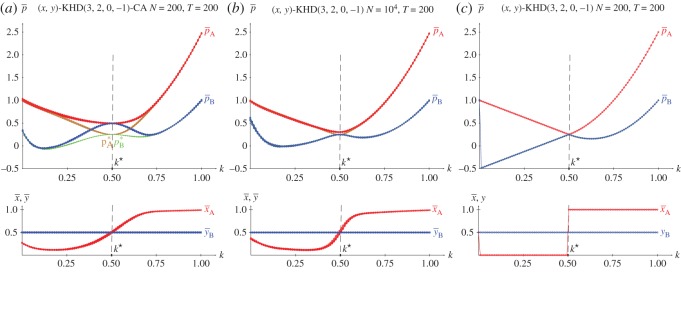
The KHD(3,2,0,−1) with variable *k* when only player A updates strategies. Five simulations at *T*=200. (*a*) Spatial simulations, (*b*) games on networks and (*c*) *y*=0.5.

**Figure 16.**
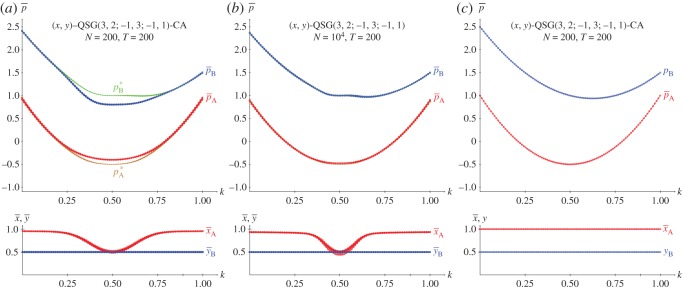
The SD(3,2,1,−1) with variable *k* when only player A updates strategies. Five simulations at *T*=200. (*a*) Spatial simulations, (*b*) games on networks and (*c*) games on networks.

**Figure 17.**
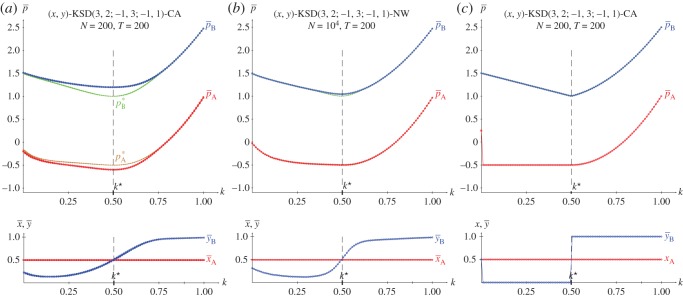
The SD(3,2,1,−1) with variable *k* when only player B updates strategies. Five simulations at *T*=200. (*a*) Spatial simulations, (*b*) games on networks and (*c*) *y*=0.5.

**Figure 18.**
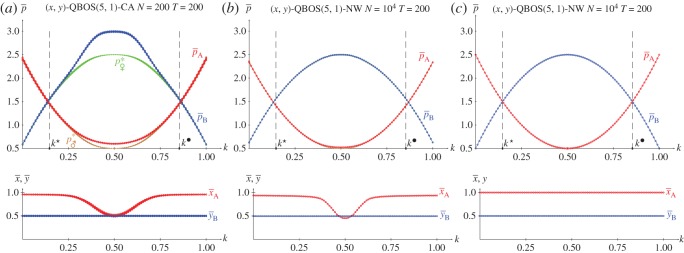
The BOS(5,1) with variable *k* when only player A updates strategies. Five simulations at *T*=200. (*a*) Spatial simulations, (*b*) games on networks and (*c*) *y*=0.5.

**Figure 19.**
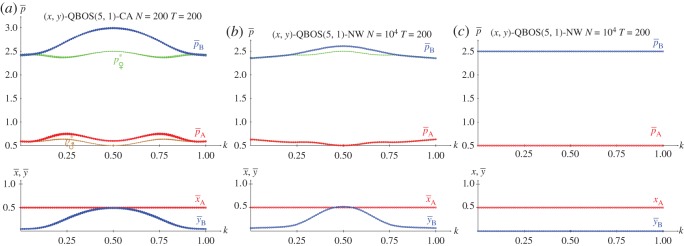
The BOS(5,1) with variable *k* when only player B updates strategies. Five simulations at *T*=200. (*a*) Spatial simulations, (*b*) games on networks and (*c*) *x*=0.5.

In this section, it is assumed that only one player type updates his strategies in the manner indicated in §3. Thus, in figures 14 and 15 only player A updates strategies in the symmetric games of PD and HD. The asymmetric games of SD and BOS are studied in figures 16–19, where both players are treated separately.

In all the figures of this section, panels *a* and *b* deal with spatial simulations and games on networks, respectively, with the initial strategy probabilities assigned at random. Panel *c*, the probability of the player that does not update his probability strategies is fixed at 0.5, instead of being assigned as random as is done with the player that updates probability strategies. Thus, panel *c* provides a kind of the theoretical reference of what is to be expected in the collective behaviour, both in spatial simulations and in games on networks.

In the mean-field analysis with partial updating, the player that does not update his probabilities will have his mean probability equal to the middle level $\frac{1}{2}$. In this scenario, the joint probability matrices, when player B is fixed to $y=\frac{1}{2}$ and player A is fixed to $x=\frac{1}{2}$, become, from equations (2.3),

5.1 $$\Pi (y=\frac{1}{2})=\frac{1}{2}\left(\begin{array}{cc} {\left(2k-1\right)}^{2}x & k-(2k-1)x\\ (1-k)+(2k-1)x & 1-{\left(2k-1\right)}^{2}x \end{array}\right)$$

and

5.2 $$\Pi (x=\frac{1}{2})=\frac{1}{2}\left(\begin{array}{cc} {\left(2k-1\right)}^{2}y & (1-k)+(2k-1)y\\ k-(2k-1)y & 1-{\left(2k-1\right)}^{2}y \end{array}\right).$$

In the KPD context of figure 14, it is $p_{A}^{\left(x,y=1/2\right)}=\frac{1}{2}(4k^{2}+4k-3))x+7-4k)$, where $(4k^{2}+4k-3)=0\to k^{⋆}=\frac{1}{2}$. Consequently, $p_{A}^{\left(x=0,y=1/2\right)}=\frac{1}{2}(7-4k)$ before *k*^★^, and *p*^(*x*=1,*y*=1/2)^_*A*_=2*k*^2^+2 after *k*^★^. As a result, the general form of the pay-off of player B, $p_{B}^{\left(x,y=1/2\right)}=\frac{1}{2}(4k^{2}-12k+5)x+4k+3)$ becomes $p_{B}^{\left(x=0,y=1/2\right)}=2k+\frac{3}{2}$ before *k*^★^, and *p*^(*x*=1,*y*=1/2)^_*B*_=2*k*^2^−4*k*+4 after *k*^★^. At $k=\frac{1}{2}$, it is $p_{A}^{\left(x=0,y=1/2\right)}=p_{A}^{\left(x=1,y=1/2\right)}=p_{B}^{\left(x=0,y=1/2\right)}=p_{B}^{\left(x=1,y=1/2\right)}=2.5$. The spatial and network simulations in figure 14 agree fairly well with these theoretical results. The discrepancies rely on the smooth transition around $k^{⋆}=\frac{1}{2}$ and on the not-exact convergence to *x*=0 and *x*=1 before and after $k^{*}=\frac{1}{2}$. As a result of the latter, at *k*=0, *p*_*A*_ slightly exceeds its theoretical value 1.5 and *p*_*B*_ slightly undervalues its theoretical value 3.5. Only small spatial effects emerge in the spatial simulations of player A (panel *a*) close to *k*^★^.

In the KHD context of figure 15, it is $p_{A}^{\left(x,y=1/2\right)}=3(2k-1)kx+1-\frac{3}{2}k$, where $2k-1=0\to k^{⋆}=\frac{1}{2}$. Consequently, $p_{A}^{\left(x=0,y=1/2\right)}=1-\frac{3}{2}k$, $p_{A}^{\left(x=1,y=1/2\right)}=6k^{2}-\frac{9}{2}k+1$. As a result, the general form of the pay-off of player B, $p_{B}^{\left(x,y=1/2\right)}=(6k^{2}k-9k+3)x+\frac{1}{2}(3k-1)$ becomes $p_{B}^{\left(x=0,y=1/2\right)}=\frac{1}{2}(3k-1)$, $p_{B}^{\left(x=1,y=1/2\right)}=6k^{2}-\frac{15}{2}k+\frac{5}{2}$. At *k*=1/2 it is, $p_{A}^{\left(x=0,y=1/2\right)}=p_{A}^{\left(x=1,y=1/2\right)}=p_{B}^{\left(x=0,y=1/2\right)}=p_{B}^{\left(x=1,y=1/2\right)}=0.25$. As reported on the PD, moderate spatial effects emerge in the spatial simulations of player A close to *k*^★^ in figure 15*a*.

The strong effect that the absence of updating capacities from one of the players exerts on the collective dynamics studied here is remarkable. Thus, figure 14*a,b* are to be compared to figures 3 and 10 regarding the PD, respectively, and figure 15*a,b* are to be compared to figures 4 and 11 regarding the HD, respectively. In any case, the intrinsic symmetry of both the PD and HD games ceases to be operative in this section, favouring player A, i.e. the player allowed to find a best response to the fixed strategies of the other player, player B, so far.

In the KSD context of figure 16, it is $p_{A}^{\left(x,y=1/2\right)}=(6k^{2}-6k+\frac{3}{2})x-\frac{1}{2}$, where $(6k^{2}-6k+\frac{3}{2})\geq 0\to x=1$, so that *p*^(*x*=1,*y*=1/2)^_*A*_=6*k*^2^−6*k*+1 and $p_{B}^{\left(x,y=1/2\right)}=(4k^{2}-6k+2)x+k-\frac{1}{2}$ becomes $p_{B}^{\left(x=1,y=1/2\right)}=4k^{2}-5k+\frac{5}{2}$. Note that the intrinsic unfairness of the SD game impedes the charity player A to overrate the beneficiary player B, even in the favourable to A scenario of figure 16. A common feature of all the simulations of this section is the non-dependence of the permanent regime of the initial configuration: The five simulations run in every frame cannot be distinguished. Thus, in the particular case of the KSD in figure 16 the outputs of the five CA and NW simulations are superimposed, so that it seems that only one has been implemented. This contrasts with the results shown in figures 6 and 12 where the five simulations may be identified before *k*^★^, although their outputs are qualitatively similar.

In the KSD context of figure 17, it is $p_{B}\left(x=1/2,y\right)=(4k^{2}-2k)y+\frac{3}{2}-k$, were $2k^{2}-k=0\to k^{⋆}=\frac{1}{2}$. Consequently, $p_{B}^{\left(x=1/2,y=0\right)}=\frac{3}{2}-k$, $p_{B}^{\left(x=1/2,y=1\right)}=4k^{2}-3k+\frac{3}{2}$; and the general form of the pay-off of player A $p_{A}\left(x=1/2,y\right)=(6k^{2}-6k+\frac{3}{2})y-\frac{3}{2}$ turns out $p_{A}\left(x=1/2,y=0\right)=-\frac{3}{2}$, *p*_*A*_(*x*=1/2,*y*)=(6*k*^2^−6*k*+1. In figure 17, the beneficiary player B overrates the charity player A, greater compared to figure 16, albeit not to a very large extent.

In the KBOS context of figure 18, it is $p_{A}^{\left(x,y=1/2\right)}=(8k^{2}-8k+2)x+\frac{1}{2}$, where 16*k*^2^−16*k*+4≥0, and consequently the best response of player A is *x*=1, which leads to $p_{A}^{\left(x=1,y=1/2\right)}=8k^{2}-8k+\frac{5}{2}$. For player B it is $p_{B}^{\left(x,y=1/2\right)}=(-8k^{2}-8k-2)x+\frac{5}{2}$, that for *x*=1 renders $p_{B}^{\left(x=1,y=1/2\right)}=-8k^{2}+8k+\frac{1}{2}$. Fairly surprisingly, these pay-offs are exactly half of those reported in the simulations of figure 13 with *k* not in its extreme values so that *x*=*y*=1, i.e. those given in equation (2.9a). In figure 18*c*, it is $\bar{x}=1$ for all *k*, also for $k=\frac{1}{2}$, but in the CA and NW simulations (figure 18*a*,*b*) it is $\bar{x}=\frac{1}{2}$ for $k=\frac{1}{2}$. Remarkably, for $k=\frac{1}{2}$ it is 8*k*^2^−8*k*+2=−8*k*^2^−8*k*−20, so that it is $p_{A}^{\left(x,y=1/2\right)}=\frac{5}{2}$ and $p_{B}^{\left(x,y=1/2\right)}=\frac{1}{2}$ regardless of *x*. As a result, there is no repercussion of being $x=\frac{1}{2}$ instead *x*=1 at $k=\frac{1}{2}$ on the actual pay-offs in the NW simulations (figure 18*b*) or in the mean-field pay-off approaches in the CA simulations (figure 18*a*). Nevertheless, in the CA simulations, spatial effects induce the increase of the actual mean pay-off of player A up to nearly ${\bar{p}}_{A}=3$. Anyhow, again in the context of this section player B overrates player A in the same wide interval of *k* of figure 13, i.e. the studied correlation mechanism favours player B in the KBOS, even if the latter is unable to update his strategies.

In the KBOS context of figure 19, it is $p_{B}\left(x=1/2,y\right)=(-8k^{2}+8k-2)y+\frac{5}{2}$, where −8*k*^2^+8*k*−2≤0 and consequently the best response of player B is *y*=0, which leads to $p_{B}\left(x=1/2,y=0\right)=\frac{5}{2}$ and $p_{A}\left(x=1/2,y=0\right)=\frac{1}{2}$. As pointed out when dealing with figure 18, for $k=\frac{1}{2}$ it is =−8*k*^2^−8*k*−2=0, so that now it is $p_{B}^{\left(x=1/2,y\right)}=\frac{5}{2}$ and $p_{A}^{\left(x=1/2,y\right)}=\frac{1}{2}$ regardless of *y* so that there is no repercussion for $y=\frac{1}{2}$ at $k=\frac{1}{2}$ on the actual pay-offs in the NW simulations or in the mean-field pay-off approaches in the CA simulations of figure 19. The spatial simulations show an odd aspect of the pay-off graphs with no explanation. Player B notably overrates player A regardless of *k* in the KBOS simulations of figure 19. This is highly expected, when in addition to the structural bias favouring player B in the KBOS, only player B is allowed to search for best responses.

## 6. Conclusion

This article studies correlated two-person games constructed from games with independent players. The games are studied in a collective manner, both in a spatially structured two-dimensional lattice and with players connected at random. Iterated games are analysed where the players interact with their nearest neighbours, and after every round each player adopts the strategy of his best paid mate neighbour for the next round. The implementation of such imitation of the best evolving rule proves to be a very useful tool to analyse the collective behaviour of two-person games via simulation.

How high correlation enables the emergence of new Nash equilibria is described. In three of the game types studied here (Prisoner’s Dilemma, Hawk and Dove, Samaritan’s Dilemma), the new Nash equilibria achieved with highly correlated games maximize the sum of the pay-offs of both players, i.e. they provide its (unique) so-called SW solution. The case of the fourth game type studied here, the Battle of the Sexes, appears to be the most challenging one in this respect because it has two SW solutions and the correlation mechanism adopted in this study tends to favour one of the players.

## Data accessibility

The Fortran code (qgames.f) used to produce all the simulations in this article as well as the makefile to run it in a Unix environment are available from the Dryad Digital Repository http://dx.doi.org/10.5061/dryad.722sg [19]. The current configuration of qgames.f produces figure 3*b*.

## References

[1] Buchanan JM 1975 The Samaritan’s dilemma. In *Altruism, morality, and economic theory* (eds ES Phelps, R Sage), p. 71. New York, NY: Russell Sage Foundation.

[2] Coate S 1995 Altruism, the Samaritan’s dilemma, and government transfer policy. *Am. Econ. Rev.* 85, 46–57.

[3] Lagerlof J 2004 Efficiency-enhancing signalling in the Samaritan’s dilemma. *Econ. J.* 114, 55–69. (doi:10.1046/j.0013-0133.2003.00176.x)

[4] Raschky PA, Schwindt M 2016 Aid, catastrophes and the Samaritan’s dilemma. *Economica* 83, 624–625. (doi:10.1111/ecca.12194)

[5] Skarbek EC 2016 Aid, ethics, and the Samaritan’s dilemma: strategic courage in constitutional entrepreneurship. *J. Inst. Econ.* 12, 371–393. (doi:10.1017/S1744137415000296)

[6] Alonso-Sanz R, Shitu H 2017 A quantum Samaritan’s dilemma cellular automaton. *R. Soc. open sci.* 4, 160669 (doi:10.1098/rsos.160669) 28680654 10.1098/rsos.160669 PMC5493896

[7] Ozdemir SK, Shimamura J, Morikoshi F, Imoto N 2004 Dynamics of a discoordination game with classical and quantum correlations. *Phys. Lett. A* 333, 218–231. (doi:10.1016/j.physleta.2004.10.055)

[8] Rasmussen E 2001 *Games and information, an introduction to game theory*. Oxford, UK: Blackwell.

[9] Binmore K 2007 *Game theory: a very short introduction*. Oxford, UK: Oxford University Press.

[10] Owen G 1995 *Game theory*. New York, NY: Academic Press.

[11] Perc M, Szolnoki A 2010 Coevolutionary games—a mini review. *Bio. Syst.* 99, 109–125. (doi:10.1016/j.biosystems.2009.10.003) 10.1016/j.biosystems.2009.10.003 19837129

[12] Szabo G, Fath G 2007 Evolutionary games on graphs. *Phys. Rep.* 446, 97–216. (doi:10.1016/j.physrep.2007.04.004)

[13] Iqbal A, Chappell JM, Abbott D 2016 On the equivalence between non-factorizable mixed-strategy classical games and quantum games. *R. Soc. open sci.* 3, 150477 (doi:10.1098/rsos.150477) 26909174 10.1098/rsos.150477 PMC4736929

[14] Binmore K 1998 *Just playing: game theory and the social contract II*. Cambridge, MA: MIT Press.

[15] Schiff JL 2008 *Cellular automata: a discrete view of the world*. New York, NY: Wiley.

[16] Eisert J, Wilkens M, Lewenstein M 1999 Quantum games and quantum strategies. *Phys. Rev. Lett.* 83, 3077–3080. (doi:10.1103/PhysRevLett.83.3077)

[17] Alonso-Sanz R 2017 On the effect of quantum noise in a quantum prisoner’s dilemma cellular automaton. *Quantum Inf. Process.* 16, 161 (doi:10.1007/s11128-017-1610-2)

[18] Alonso-Sanz R 2014 Variable entangling in a quantum battle of the sexes cellular automaton. In *ACRI-2014*. Lecture Notes in Computer Science, no. 8751, pp. 125–135. Basel, Switzerland: Springer International Publishing.

[19] Alonso-Sanz R 2017 Data from: Spatial correlated games Dryad Digital Repository. (http://dx.doi.org/10.5061/dryad.722sg) 10.1098/rsos.171361 PMC5717695 29291120

